# Detecting Epileptic Seizure from Scalp EEG Using Lyapunov Spectrum

**DOI:** 10.1155/2012/847686

**Published:** 2012-03-05

**Authors:** Truong Quang Dang Khoa, Nguyen Thi Minh Huong, Vo Van Toi

**Affiliations:** ^1^Biomedical Engineering Department, nternational University of Vietnam National Universities, Ho Chi Minh City, Vietnam; ^2^Faculty of Applied Science, University of Technology of Vietnam National Universities, Ho Chi Minh City, Vietnam

## Abstract

One of the inherent weaknesses of the EEG signal processing is noises and artifacts. To overcome it, some methods for prediction of epilepsy recently reported in the literature are based on the evaluation of chaotic behavior of intracranial electroencephalographic (EEG) recordings. These methods reduced noises, but they were hazardous to patients. In this study, we propose using Lyapunov spectrum to filter noise and detect epilepsy on scalp EEG signals only. We determined that the Lyapunov spectrum can be considered as the most expected method to evaluate chaotic behavior of scalp EEG recordings and to be robust within noises. Obtained results are compared to the independent component analysis (ICA) and largest Lyapunov exponent. The results of detecting epilepsy are compared to diagnosis from medical doctors in case of typical general epilepsy.

## 1. Introduction

Since the discovery of the human electroencephalographic (EEG) signals by Hans Berger in 1923, the EEG has been the most commonly used instrument for clinical evaluation of brain activity, classification epileptic seizures or no epilepsy, schizophrenia, sleep disorder, mental fatigue, and coma. 

There were many researches on EEG in the world. An EEG signal is a measurement of currents that flow during synaptic excitations of the dendrites of many pyramidal neurons in the cerebral cortex. When brain cells (neurons) are activated, the synaptic currents are produced within the dendrites. This current generates a secondary electrical field over the scalp measurable by EEG systems. They are captured by multiple-electrode EEG machines either from inside the brain, over the cortex under the skull, or certain locations over the scalp and can be recorded in different formats. 

Today, the epilepsy is important problem in the public healthy and everyone should be specially interested in it because its effects are influenced on the life's qualities, study, and working abilities, falling in line with society badly. Epilepsy is the most common neurological disorder, second only to stroke. Nearly 60 million people worldwide are diagnosed with epilepsy whose hallmark is recurrent seizures [1]. Some 35 million have no access to appropriate treatment. This is either because services are nonexistent or because epilepsy is not viewed as a medical problem or a treatable brain disorder.

Most traditional analyses of epilepsy, based on the EEG, are focused on the detection and classification of epileptic seizures. Among them, the best method of analysis is still the visual inspection of the EEG by a highly skilled electroencephalographer. However, with the advent of new signal processing methodologies based on the mathematical theory, there has been an increased interest in the analysis of the EEG for prediction of epileptic seizures.

To detect spike, Gotman and Wang [2] defined 5 states (active wakefulness, quiet wakefulness, desynchronized EEG, phasic EEG, and slow EEG) and designed one method for automatic state classification. Then, they designed procedures for identification of nonepileptic transients (eye blinks, EMG, alpha, spindles, vertex sharp waves) by measuring parameters such as relative amplitude, sharpness, and duration of EEG waves. This method is sensitive to various artifacts. In attempts to overcome that artifacts, Dingle et al. [3] developed a multistage system to detect the epileptiform activities from the EEGs. In another approach, Glover et al. [4] used a correlation-based algorithm that was attempted to reduce the muscle artefacts in multichannel EEGs. So, approximately 67% of the spikes can be detected. By incorporating both multichannel temporal and spatial information, and including the electrocardiogram, electromyogram, and electrooculogram information into a rule-based system [5], a higher detection rate was achieved. Artificial neural networks (ANNs) have been used for seizure detection by many researchers [6, 7]. To predict epilepsy, Zhu and Jiang [8] tracks the time evolution of the slow wave energy bigger than some preset threshold from scalp EEGs. The results from four generalized epileptic patients demonstrate that pre-seizure transition phases of several minutes can be identified clearly by their linear predictor. Among recent works, time-frequency (TF) approaches effectively use the fact that the seizure sources are localized in the time-frequency domain. Most of these methods are mainly for detection of neural spikes [9] of different types. The methods are especially useful since the EEG signals are statistically nonstationary.

One of tendencies to predict seizure is nonlinear methods. The brain is assumed to be a dynamical system, since epileptic neuronal networks are essentially complex nonlinear structures and a nonlinear behavior of their interactions is, thus, expected. So, these methods have substantiated the hypothesis that quantification of the brain's dynamical changes from the EEG might enable prediction of epileptic seizures, while traditional methods of analysis have failed to recognize specific changes prior to seizure [1]. These include reduction in correlation integrals during the ictal state (Lerner, 1996) [10] and decrease in signal complexity during seizures. In 1998, Le Van Quyen et al. [11] contributed a new measurement in prediction seizure which they called “correlation density”. Then, this group has introduced newer nonlinear techniques, such as the “dynamical similarity index” [12, 13], which measures similarity of EEG dynamics between recordings taken at distant moments in time. Jerger et al. [14] and Jouny et al. [15] used two methods, one of which, Gabor atom density, estimates intracranial EEGs in terms of synchrony and complexity. In another other approach, Esteller et al. [16] introduced parameter of average energy of EEG signal. They demonstrated that when seizure happens, there were bursts of complex epileptiform activity, delta slowing, subclinical seizures, and gradual increases in energy in the epileptic focus. Harrison et al. [17] measured the amount of energy in EEG signal and its averaged power within moving windows. 

Iasemidis introduced ideas of chaotic in predicting seizure. In 1988 and 1990, Iasemedis et al. [18] published the first of a number of prominent articles describing another nonlinear measure for predicting seizures, primarily the largest Lyapunov exponent, for characterizing intracranial EEG recordings [19]. The lowest values of Lyapunov occur during the seizure but they are still positive denoting the presence of a chaotic attractor. Then, this group introduced an efficient version of the Largest Lyapunov Exponent (Lmax) named *Short-Term Maximum Lyapunov Exponent *(STLmax) and proved the relationship between the temporal evolution of Lmax and the development of epileptic seizures [20].

Most of these studies for prediction of epilepsy are based on intracranial EEG recordings. These methods faced main challenge. This is hazardous to the patient, especially the children. The scalp EEG is the most popular recording in Hospitals. But the scalp signals are more subject to environmental noise and artifacts than the intracranial EEG, and the meaningful signals are attenuated and mixed in their way via soft tissue and bone. So, the tradition methods such as the Kolmogorov entropy or the Lyapunov exponents, may be affected by the after mentioned two difficulties and, therefore, they may not distinguish between slightly different chaotic regimes of the scalp EEG [21]. There are many researchers to be interested in this problem. They tried to applied tradition nonlinear measurement to scalp EEG. This is the approach followed by Hively and Protopopescu [22]. They proposed a method based on the *phase-space dissimilarity measures *(PSDMs) for forewarning of epileptic events from scalp EEG. Iasemidis et al. [23], using the spatiotemporal evolution of the short-term largest Lyapunov exponent, demonstrated that minutes or even hours before seizure, multiple regions of the cerebral cortex progressively approach a similar degree of chaoticity of their dynamical states. They called it *dynamical entrainment. *This method has also been shown to work well on scalp-unfiltered EEG data for seizure predictability. In 2006, a research group of Saeid Sanei developed a novel approach to quantify the dynamical changes of the brain using the scalp EEG by means of an effective block-based blind source separation (BSS) technique to separate the underlying sources within the brain to overcome problems of noises and artifacts. Their methods are promising but their results also faced noises and artifact [1].

 Here, we are only interested in applying the Lyapunov exponent for scalp EEG to predict epilepsy. Like previous methods, the main problem to apply the Lyapunov exponents for scalp EEG is noises. We executed combined ICA method and Lyapunov exponent by Rosenstein. In addition, we also find improvements of Lyapunov spectrum in estimating the Lyapunov exponent so that it can be more robust, especially with respect to the presence of noise in the EEG.

 This paper is organized as follows. In Section 2, we describe the algorithms for filtering, estimating that the Lyapunov exponent, especially Lyapunov spectrum, considered as an optimization model for estimating Lyapunov exponents is presented. In Section 3, the EEG recording procedure is explained and the results are compared with the other methods. Conclusions are provided in Section 4.

## 2. Materials and Methods

### 2.1. Materials

The experimental data were derived from the Hospital 115 in Ho Chi Minh City, Vietnam using a Galileo EEG machine (EBNEURO, Italy) and divided into three groups: seizures (8 files), brain function disorder due to epilepsy or transform (7 files), nonseizure (15 files).

### 2.2. Preprocessing

Frequencies of EEG signals are less than 100 Hz. In addition, most recordings present a 50-Hz frequency component contaminating several electrodes. Therefore, the signals need to be lowpass filtered to eliminate this frequency component and other high-frequency components generally produced by muscular activity. A Butterworth filter of order 10 with a cutoff frequency of 45 Hz is used [1]. Within this range of frequencies, we still have the complete information about the signals. 

### 2.3. Independent Component Analysis (ICA) [24, 25]

After the preprocessing step, the scalp EEG is still contaminated by noise and artifacts such as eye blinks. Independent component analysis (ICA) is an effective method for removing artifacts, especially eye blinks, and separating sources of the brain signals from these recordings. ICA methods are based on the assumptions that the signals recorded on the scalp are mixtures of time courses of temporally independent cerebral and artifactual sources, that potentials arising from different parts of the brain, scalp, and body are summed linearly at the electrodes, and that propagation delays are negligible.

### 2.4. Lyapunov Exponents

The EEG recorded from one site is inherently related to the activity at other sites. This makes the EEG a multivariable time series. Generally, an EEG signal can be considered as the output of a nonlinear system, which may be characterized deterministically. Methods for calculating these dynamical measures from experimental data have been published [26]. Among them, Lyapunov exponent is one of parameters to measure chaos of a nonlinear dynamical system and characterizes the spatiotemporal dynamics in electroencephalogram (EEGs) time series recorded from patients with temporal lobe epilepsy. Wolf et al. [27] proposed the first algorithm for calculating the largest Lyapunov exponent. But the Wolf algorithm only estimates the largest Lyapunov exponent and the first few nonnegative Lyapunov exponents, not the whole spectrum of exponents. It is sensitive to noises of time series as well as to the degree of measurement or unreliable for small data sets. So, Iasemidis et al. presented algorithm of estimating the short-term largest Lyapunov exponent, which is a modified version of the program proposed of Wolf. This modification is necessary for predicting seizure (small data segments of epileptic data). Besides, there were many improvements in estimating the Lyapunov exponent of many researchers in the world such as Eckmann et al. [28], Brown et al. [29], and Rosenstein et al. [30]. Here, we also used the algorithm of Rosenstein because of its advantages. The Rosenstein algorithm is fast, easy to implement, and robust to changes in the following quantities: embedding dimension, size of data set, reconstruction delay, and noise level. Furthermore, one may use the algorithm to calculate simultaneously the correlation dimension. Thus, one sequence of computations will yield an estimate of both the level of chaos and the system complexity.

### 2.5. The Rosenstein Algorithm [30]

The first step of our approach involves reconstructing the attractor dynamics from a single time series. We use the method of delays since one goal of our work is to develop a fast and easily implemented algorithm. The reconstructed trajectory, X, can be expressed as a matrix where each row is a phase-space vector. That is,

- vector X_*i*_ in phase space:

(1) $$x_{i}=\left(x\left(t_{i}\right)\right),\;x\left(t_{i}+\tau \right),\ldots ,x\left(t_{i}+\left(p-1\right)*\tau \right),$$

where *τ* is the *lag *or *reconstruction delay*, *p* is the *embedding dimension*, and *t*_*i*_ ∈ [1, *T* − (*p* − 1)*τ*]

- from the definition of *λ*_1_ given in theory *d*(*t*) = *C*exp⁡(*λ*1*t*), we assume that the *j*th pair of nearest neighbors diverge proximately at a rate given by the largest Lyapunov exponent:

(2) $$d_{j}\left(i\right)\approx C_{j}e^{\left(i\cdot \Delta t\right)},$$

where *C*_*j*_ is the initial separation. By taking the logarithm of both sides of (2), we have

(3) $$\ln d_{j}\left(i\right)\approx \ln C_{j}+\lambda_{1}\left(i\cdot \Delta t\right).$$

Equation (3) represents a set of approximately parallel lines (for *j* = 1,2,…, *m*), each with a slope roughly proportional to *λ*_1_. The largest Lyapunov exponent is easily and accurately calculated using a least-squares fit to the “average” line defined by

(4) $$y\left(i\right)=\frac{1}{\Delta t}\langle \ln d_{j}\left(i\right)\rangle ,$$

where 〈·〉 denotes the average over all values of *j*. This process of averaging is the key to calculating accurate values of *λ*1 using small, noisy data sets. Note that in (3), *C*_*j*_ performs the function of normalizing the separation of the neighbors, but as shown in (4), this normalization is unnecessary for estimating *λ*_1_. By avoiding the normalization, the current approach gains a slight computational advantage over the method by Sato et al. [31].

#### 2.5.1. The Lyapunov Spectrum [32]

Another way to view Lyapunov exponents is the loss of predictability as we look forward in time. If we assume that the true starting point *x*0 of a time series is possibly displaced by an *ε*, we know only the information area *I*0 about the starting point. After some steps, the time series is in the information area at time *t*, *It*. The information about the true position of the data decreases due to the increase of the information area. Consequently, we get a bad predictability. The largest Lyapunov exponent can be used for the description of the average information loss; *λ*1 > 0 leads to bad predictability [32]. While there is a method which is applicable to many dimensional chaos to extract physical quantities from experimentally obtained irregular signals is Lyapunov spectrum [33]. It estimates the spectrum of several Lyapunov exponents (including positive, zeros, and even negative ones). This is necessary for quantifing many physical quantities, especially for complicating EEG signals. Besides, in EEG processing, a main problem is noises and artifacts. There are many researches about processing EEG, especially removing noises to predict epilepsy. But most of reports only solved part of problems and faced some difficulties. Here, we will describe a method of Lyapunov spectrum which is shown to behave well in the perturbation of certain parameter values, but slightly sensitive in the presence of noise, good accuracy with great easy. It is suitable to prediction seizure. 

Let us consider an observed trajectory *x*(*t*), which can be considered as a solution of a certain dynamical system:

(5) $$\dot{x}=F\left(x\right)$$

defined in a *d*-dimensional phase space. On the other hand, the evolution of a tangent vector *ξ* in a tangent space at *x*(*t*) is represented by linearizing (5):

(6) $$\dot{\xi }=T\left(x\left(t\right)\right)\cdot \xi ,$$

where *T* = *DF* = ∂*F*/∂*x* is the Jacobian matrix of *F*. The solution of the linear nonautonomous (6) can be obtained as

(7) $$\xi \left(t\right)=A^{t}\xi \left(0\right),$$

where *A* ^*t*^ is the linear operator which maps tangent vector *ξ*(0) to *ξ*(*t*). The mean exponential rate of divergence of the tangent vector *ξ* is defined as follows:

(8) $$\lambda \left(x\left(0\right),\xi \left(0\right)\right)=\underset{t\to \infty }{\lim}\frac{1}{t}\ln\frac{||\xi \left(t\right)||}{||\xi \left(0\right)||},$$

where ||⋯|| denotes a norm with respect to some Riemannian metric. Furthermore, there is a *d*-dimensional basis {*e*_*i*_}  of *ξ*(0), for which *λ* takes values *λ*_*i*_(*x*(0)) = *λ*(*x*(0), *e*_*i*_). These can be ordered by their magnitudes *λ*_1_ ≥ *λ*_2_ ⋯ ≥*λ*_*n*_, and are the spectrum of Lyapunov characteristic exponents. These exponents are independent of *x*(0) if the system is ergodic.

**Algorithm 1**

Let {*x*_*j*_} (*j* = 1,2,…) denote a time series of some physical quantity measured at the discrete time interval Δ*t*, that is, *x*_*j*_ = *x*(*t*_0_ + (*j* − 1)Δ*t*). Consider a small ball of radius *ε* centered at the orbital point *x*_*j*_, and find any set of points {*x*_*k*_*i*__} (*i* = 1,2,…, *N*) included in this ball, that is,

(9) $$\left\{y^{i}=\left\{x_{k_{i}}-x_{j}\;|\;\;||x_{k_{i}}-x_{j}||\leq \epsilon \right\}\right\},$$

where *y* ^*i*^ is the displacement vector between *x*_*k*_*i*__ and *x*_*j*_. We used a usual Euclidean norm defined as follows: ||*w*|| = (*w*_1_^2^ + *w*_2_^2^ + ⋯+*w*_*d*_^2^)^1/2^ for some vectors *w* = (*w*_1_, *w*_2_,…, *w*_*d*_). After the evolution of a time interval *τ* = *m*Δ*t*, the orbital point *x*_*j*_ will proceed to *x*_*j*+*m*_ and neighboring points {*x*_*k*_*i*__}  *to*  {*x*_*k*_*i*_+*m*_}. The displacement vector *y* ^*i*^ = *x*_*k*_*j*__ − *x*_*j*_ is thereby mapped to

(10) $$\left\{z^{i}\right\}=\left\{x_{k_{i}+m}-x_{j+m}\;|\;\;||x_{k_{i}}-x_{j}||\leq \epsilon \right\}.$$

If the radius *ϵ* is small enough for the displacement vectors {*y* ^*i*^} and {*z* ^*i*^} to be regarded as good approximation of tangent vectors in the tangent space, evolution of *y* ^*i*^  to  *z* ^*i*^ can be represented by some matrix *A*_*j*_, as follows:

(11) $$z^{i}=A_{j}y^{i}.$$

The matrix *A*_*j*_ is an approximation of the flow map *A* ^*t*^ at *x*_*j*_ in (7). Let us proceed to the optimal estimation of the linearized flow map *A*_*j*_ from the data sets {*y* ^*i*^}  and  {*z* ^*i*^}. A plausible procedure for optimal estimation is the least-square-error algorithm, which minimizes the average of the squared error norm between *z* ^*i*^ and *A*_*j*_ *y* ^*i*^ with respect to all components of the matrix *A*_*j*_ as follows:

(12) $$\underset{A_{j}}{\min}\;S=\underset{A_{j}}{\min}\frac{1}{N}\overset{N}{\underset{i=1}{\sum }}{||z^{i}-A_{j}y^{i}||}^{2}.$$

Denoting the (*k*, *l*) component of matrix *A*_*j*_ and applying condition (12), one obtains *d* × *d* equations to solve ∂*S*/∂*a*_*kl*_(*j*) = 0. One will easily obtain the following expression for *A*_*j*_:

(13) $$\begin{array}{c} A_{j}V=C,\;\left(V_{kl}\right)=\frac{1}{N}\overset{N}{\underset{i=1}{\sum }}y^{ik}y^{il},\\ \left(C_{kl}\right)=\frac{1}{N}\overset{N}{\underset{i=1}{\sum }}Z^{ik}y^{il}, \end{array}$$

where *V* and *C* are *d* × *d* matrices, called covariance matrices, and và *y* ^*ik*^ and *Z* ^*ik*^ are the k components of vectors *y* ^*i*^ and *z* ^*i*^, respectively. If *N* ≥ *d* and there is no degeneracy, (13) has a solution for *a*_*kl*_(*j*).

Now that we have the variational equation in the tangent space along the experimentally obtained orbit; the Lyapunov exponents can be computed as

(14) $$\lambda_{i}=\underset{n\to \infty }{\lim}\frac{1}{n\tau }\overset{n}{\underset{j=1}{\sum }}\ln||A_{j}e_{i}^{j}||,$$

for *i* = 1,2,…, *d*, where *A*_*j*_ is the solution of (13), and {*e*_*i*_^*j*^} (*i* = 1,2,…, *d*) is a set of basis vectors of the tangent space at *x*_*j*_. In the numerical procedure, choose an arbitrary set {*e*_*i*_^*j*^}. Operate with the matrix *A*_*j*_ on {*e*_*i*_^*j*^}, and renormalize *A*_*j*_ *e*_*i*_^*j*^ to have length 1. Using the Gram-Schmidt procedure, maintain mutual orthogonality of the basis. Repeat this procedure for *n* iterations and compute (14). The advantage of the present method is now clear, since we can deal with arbitrary vectors in a tangent space and trace the evolution of these vectors. In this method, these vectors are not restricted to observed data points, in contrast with the conventional methods. The feature allows us to compute all exponents to good accuracy with great easy.

## 3. Results and Discussions

Signals are firstly preprocessed by Butterworth filter of order 10 with a cutoff frequency of 45 Hz to remove noise 50 Hz and high-frequency components. Filtered signals were then analyzed by Independent Component Analysis (ICA) to get main components for comparison purposes. Quantifying the changes in the brain dynamics was carried out by nonlinear methods such as estimating the largest Lyapunov exponent *λ*_1_ and the Lyapunov spectrum was also used to evaluate chaotic behavior of scalp EEG recordings.

**Figure 1:**
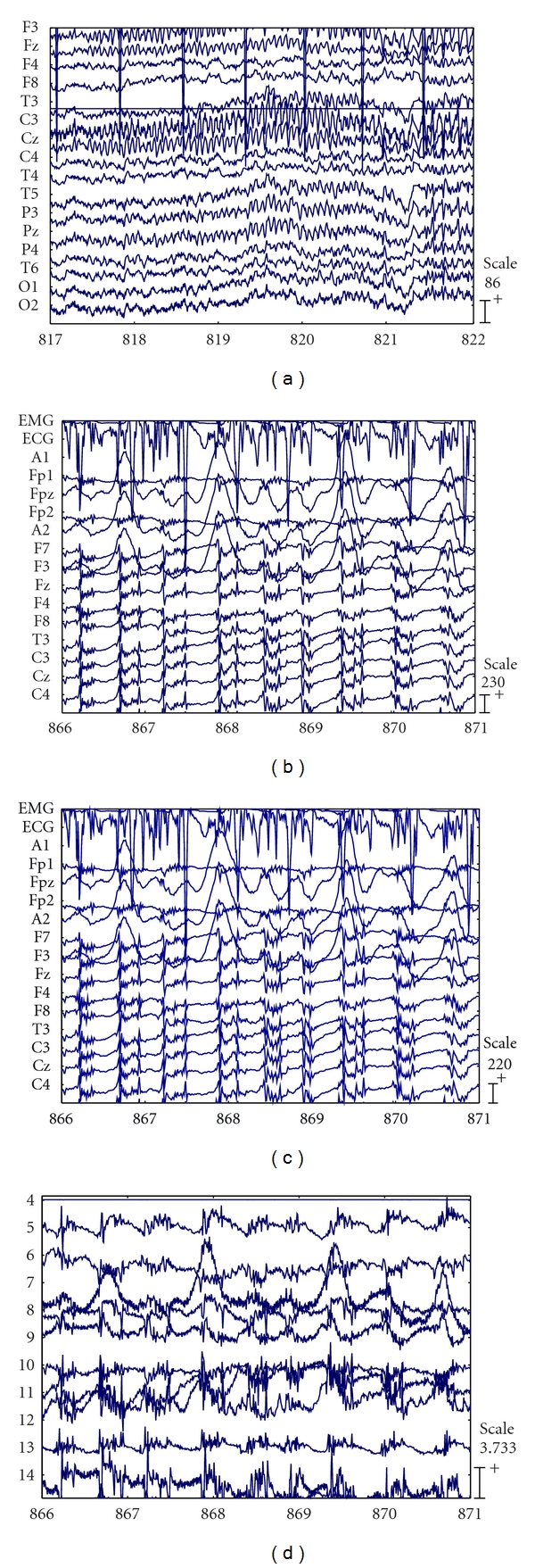
A scalp EEG recording of 21 minutes containing a general epilepsy. (a) The 5-second EEG segment at the preictal of frontal seizure was recorded by the scalp electrodes before removing noises. (b) EEG signal (5 s) during the seizure. (c) The result of (b) after being filtered by Butterworth filter of order 10 with a cutoff frequency of 45 Hz. (d) The signals obtained after applying the proposed ICA algorithm to the same segment (c).

Figures 1(a)–1(d) show the results obtained for a scalp EEG recording of 21 minutes containing a general epilepsy. In Figure 1(a), the 5-second EEG segment at the preictal of frontal seizure was recorded by the scalp electrodes before removing noises. At second 817, there are series of high-frequency, repetitive spikes, polyspike-slow waves. The preseizure was clearly discernible in the scalp electrodes, around second 817, and the seizure state lasted until the second 871 (Figure 1(b)). The signals are contaminated by noises and artifacts but the seizure is discernible.  Figure 1(c) is result of Figure 1(b) after being filtered by Butterworth filter of order 10 with a cutoff frequency of 45 Hz.  Figure 1(d) shows the signals obtained after applying the proposed ICA algorithm to the same segment in Figure 1(c). The IC4, IC9, and IC10 are sources of noise EEG while the seizure components are in remaining ICs. 

**Figure 2:**
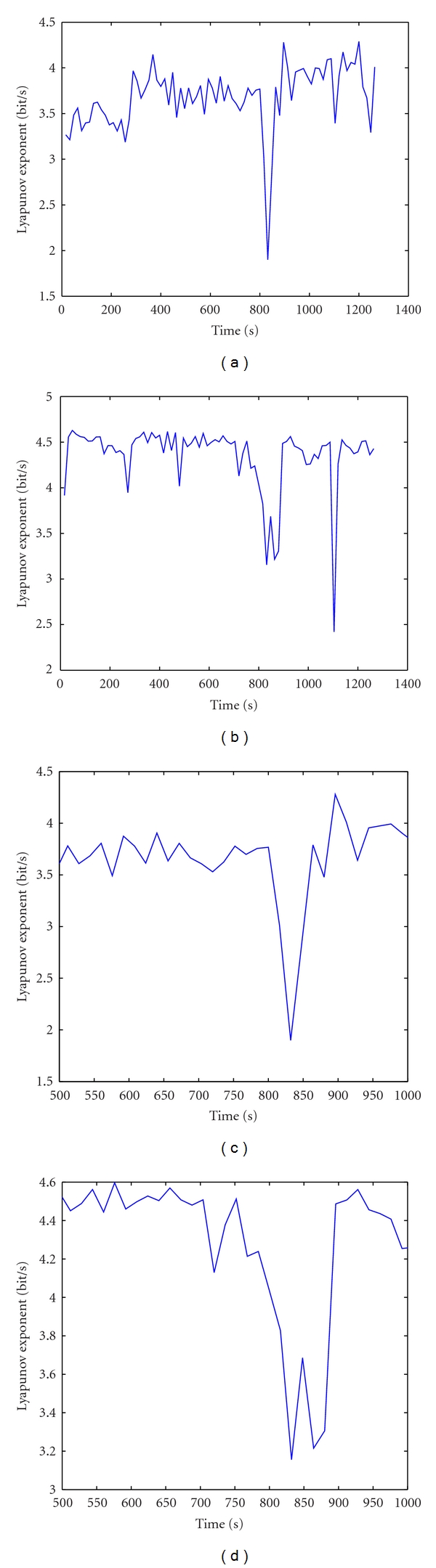
The Lyapunov exponent's profiles over time of IC6 and IC7. (a) and (b) are the largest Lyapunov exponent's profiles over time of IC6 and IC7. (c) and (d) are the largest Lyapunov exponent's profiles of IC6 and IC7 obtained by observing the Lyapunov profiles from second 500 to second 1000.

Figures 2(a) and 2(b) are Lyapunov profiles over time of IC6 and IC7. Both these ICs showed that, during the seizure from second 817 to 871, the Lyapunov exponents start decreasing, and at about second 847, Lyapunov exponents drop to minimum. The seizure can easily be detected from the lowest values of Lyapunov exponent. It is period of second 817 to 871. These results are suitable to points recorded in Figure 1. Besides, Figures 2(c) and 2(d), the Lyapunov profiles of IC6 and IC7 obtained by observing the Lyapunov profiles from second 500 to second 1000, show that *λ*_1_ starts decreasing approximately 2 mins before the onset of seizure. Therefore, the Lyapunov profiles of ICs after be analyzed Independent Component can help doctors not only to detect but also to predict early seizures for 2 minutes before the seizure occurs.

**Figure 3:**
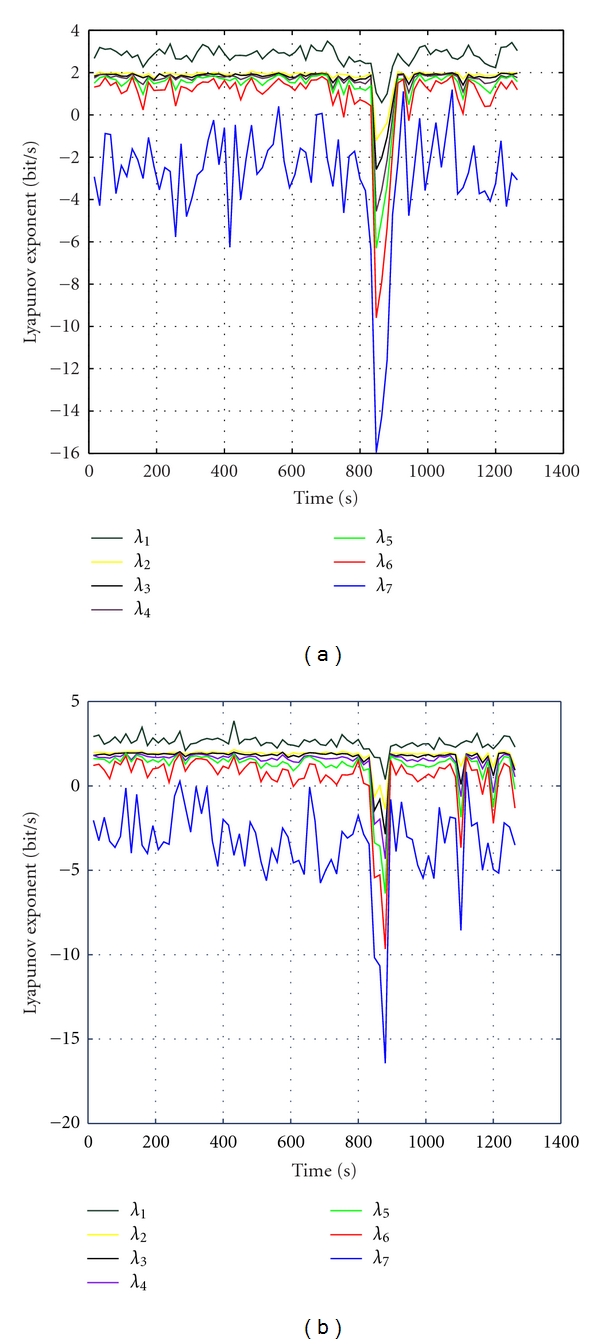
The Lyapunov spectrum profiles of IC6 and IC7 of the same data.

 Figures 3(a) and 3(b) are the Lyapunov spectrum profiles of IC6 and IC7 of the same data. The maximum drop of Lyapunov coefficients occurs around 847, where seizure happens. It means that Lyapunov spectrum can be used to detect seizure accurately. Moreover, observing a period of 5 minutes of the preseizure-seizure, we can see that all the Lyapunov coefficients decrease approximately two minutes before the seizure happened. This helps the doctors to predict seizure. These results clearly show that the proposed ICA algorithm successfully separates the seizure signal (long before the seizure) from the rest of the sources, noise, and artifacts within the brain. Both the largest Lyapunov exponents and Lyapunov spectrum can be combined with ICA methods to quantify the changes in brain dynamic for diagnosing epilepsy and have brought good results.

**Figure 4:**
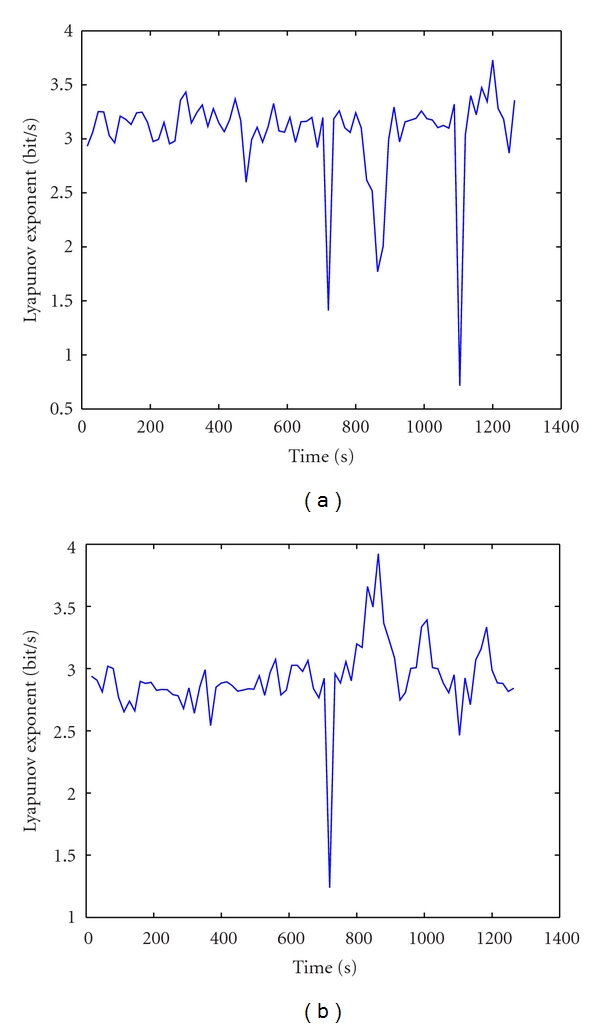
The largest Lyapunov exponent's profiles of channels 8 and 11, respectively.

Figures 4(a) and 4(b) are the Lyapunov profiles of the channels 8 and 11, respectively. Most channels show a minimum drop in the value of *λ*_1_  around second 720, while preseizure-seizure onset interval which occurs at second 817 to second 871 has maximum peaks of the Lyapunov coefficient. Therefore, none of the channels is able to detect and predict seizure. Moreover, the scalp EEG after filtering 0.5–45 Hz was contaminated by a high-frequency activity that causes fluctuations of for the entire recording. So, estimating only the largest Lyapunov coefficient of scalp EEG without ICA showed that mentioned features cannot detect the seizure.

**Figure 5:**
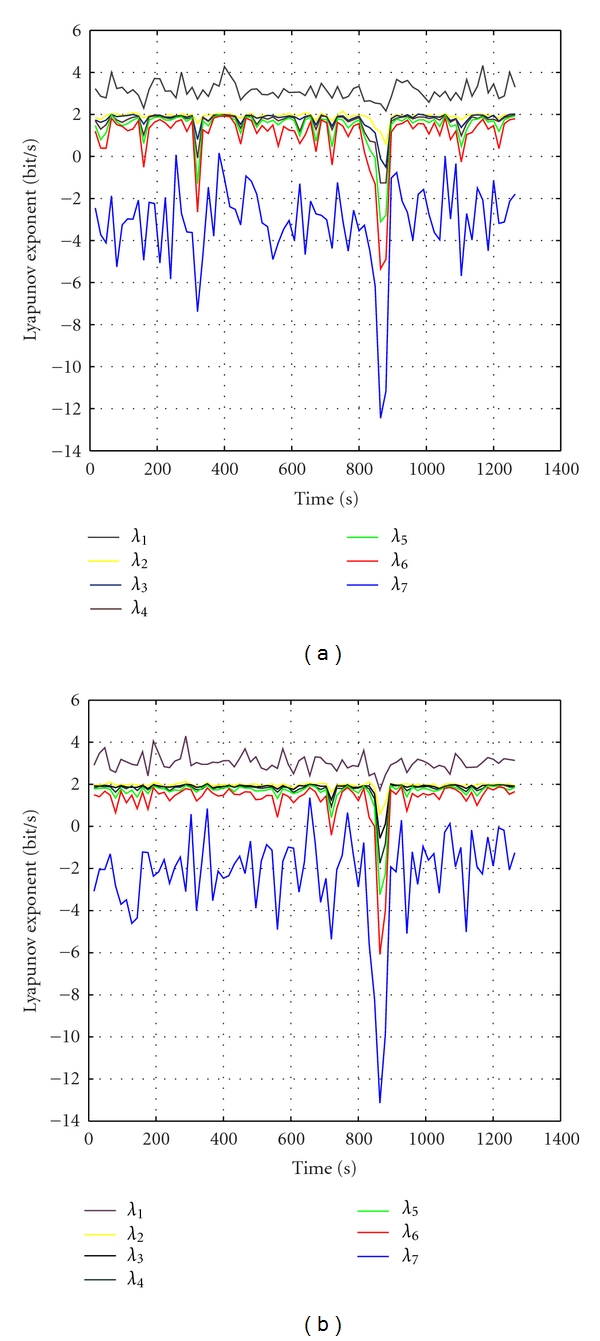
The Lyapunov spectrum of channels 8 and 11.

 The detection could be improved by examining the Lyapunov spectrum with other *λ* parameters. Figures 5(a) and 5(b) are Lyapunov spectrums of the channels 8 and 11 after being filtered 0–45 Hz. The Lyapunov coefficients start decreasing around second 800 and reach minimum around second 890. There is the interval in that pre-seizure and onset seizure occur. Moreover, minimum drop of *λ*_1_ is not as clear as these of other Lyapunov coefficients. This result showed that the Lyapunov spectrum can detect seizure for noiseful scalp EEG when the largest Lyapunov coefficient method cannot. This is an advantage for processing scalp EEG in practical cases in Hospital.

**Figure 6:**
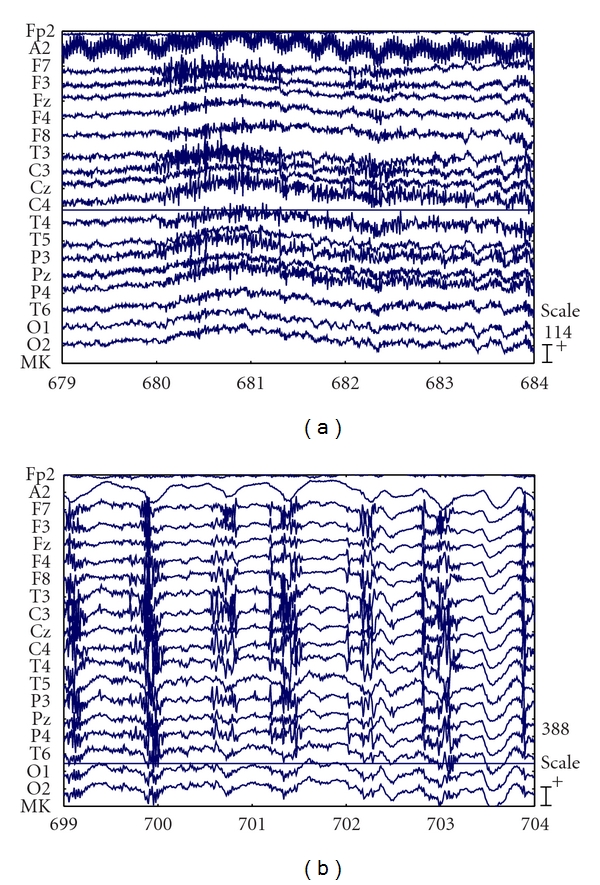
The scalp EEG recordings of 21 minutes containing a general epilepsy. (a) The 5-second EEG segment at the pre-seizure of frontal seizure. (b) EEG signal (5 s) during the seizure.

Figures 6(a) and 6(b) show scalp EEG recordings of 21 minutes containing a general epilepsy. In Figure 6(a), the 5-second EEG segment at the pre-seizure of frontal seizure was recorded by the scalp electrodes before removing noises from second 679 to 684. We can see the complexity of the signal decreased and the shape of sin. Then the period of seizure occurs with signs of paroxysmal depolarization, and the waveform becomes much more complicated. Seizure ends at second 724. These signals are filtered by Butterworth filter of order 10 with a cutoff frequency of 45 Hz and then are analysed by ICA method to separate the seizure signal (long before the seizure) from the rest of the sources, noise, and artifacts within the brain. While ICs bring seizure signs, the Lyapunov exponents are estimated.

**Figure 7:**
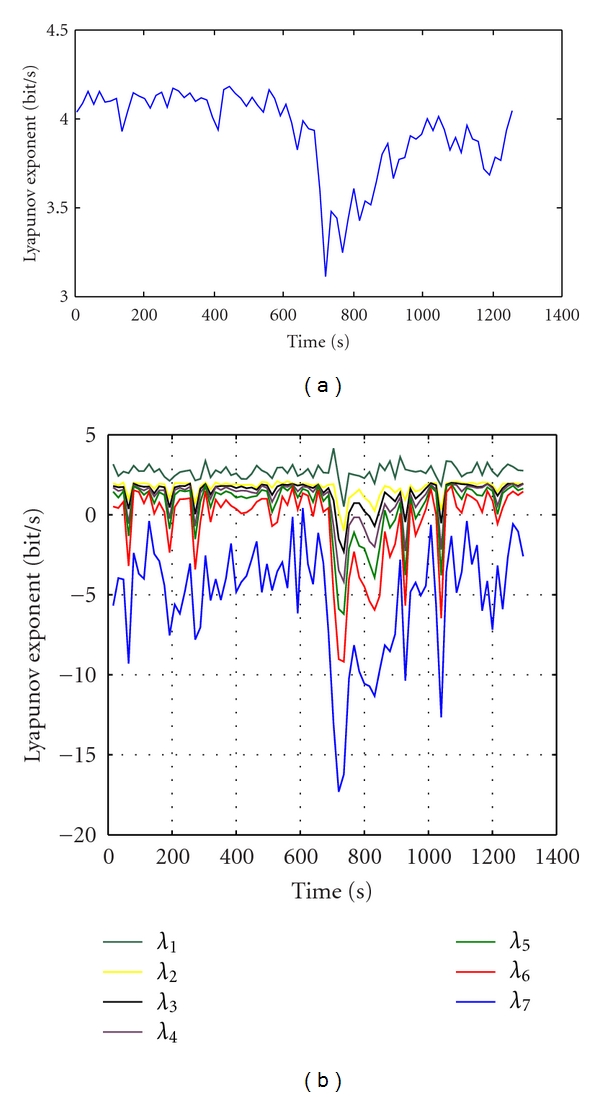
The changes in the Lyapunov exponent for IC5. (a) The smoothed *λ*_1_ of IC5. (b) The Lyapunov spectrum of IC5.

Figures 7(a) and 7(b) illustrate the changes in the smoothed *λ*_1_ for IC5 brings seizure signal obtained by the lagest Lyapunov exponent and Lyapunov spectrum method, respectively. *λ*_1_ starts decreasing at second 600, approximately 2 minutes before the onset of seizure, and drops minimum around second 725. The experiment results showed that ICA algorithm successfully separates the seizure signal and the combination of ICA and the Lyapunov exponent method can help the doctors not only detect but also predict the epilepsy. This is an effective combination not only in removing the noises for processing the EEG signal but also quantifying the changes of brain changes as well.

**Figure 8:**
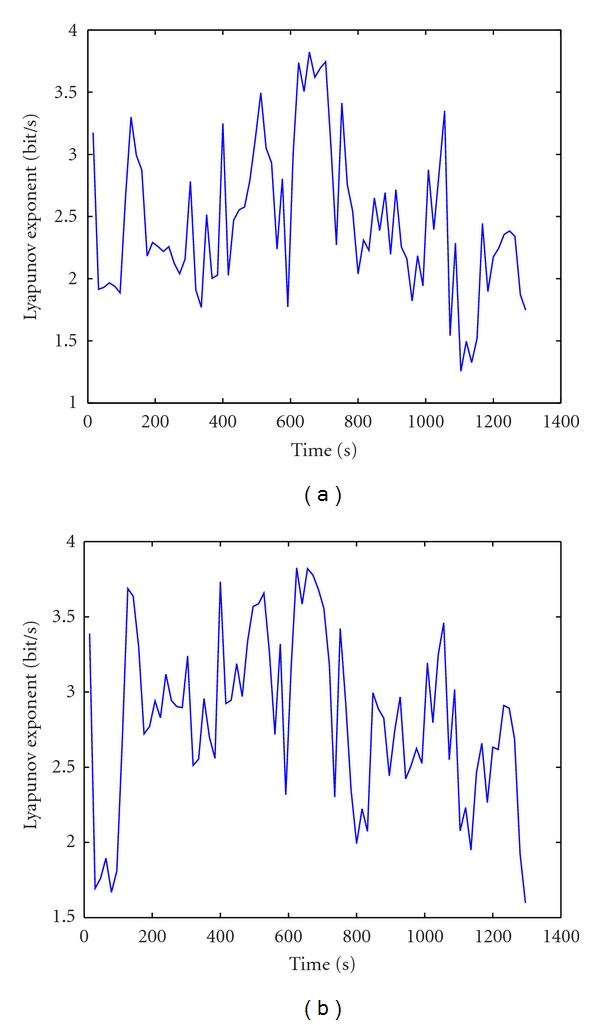
The largest Lyapunov exponent's profiles of channels 9 and 10.

Figures 8(a) and 8(b) are Lyapunov profiles of channels 9 and 10. The values *λ*_1_ have large fluctuations that can be due to the presence of the noises and artifacts. More over, there are no clear drops of *λ*_1_ before, in and after seizure happens. It means that the maximum Lyapunov is sensitivity to noises and it cannot detect epilepsy with quite noisy EEG. This can be caused by the description of the average information loss of *λ*_1_. As mentioned previously, the detection could be improved by examining the Lyapunov spectrum with other *λ* parameters.

**Figure 9:**
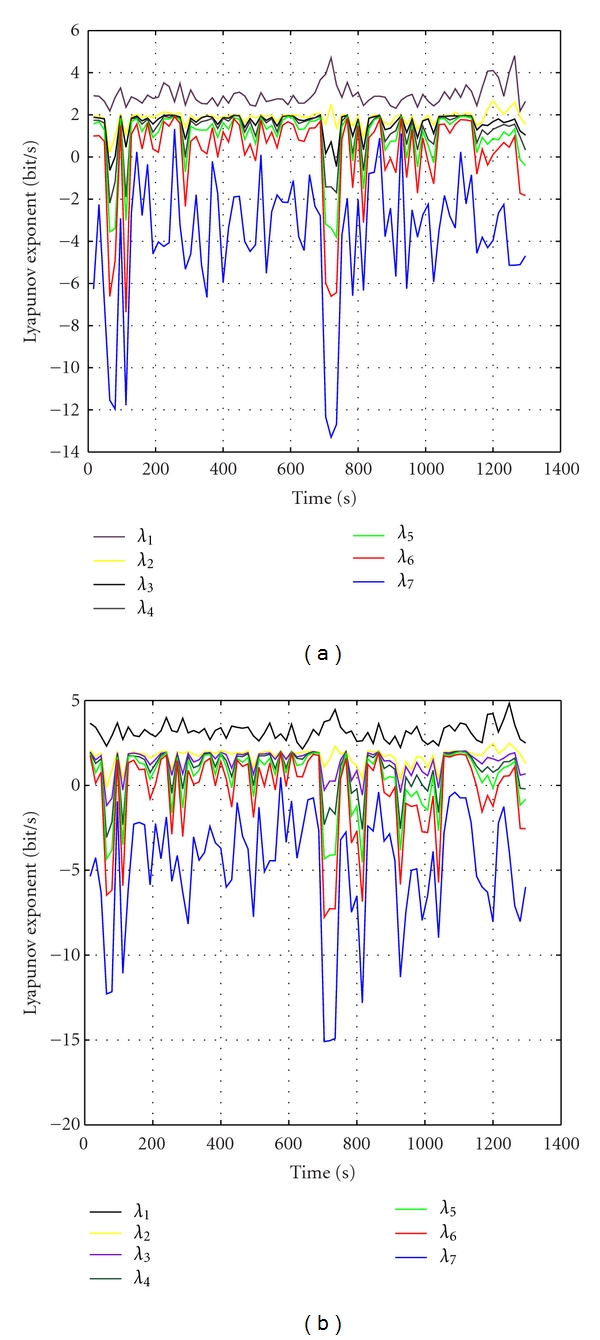
The Lyapunov spectrum of channels 9 and 10.

Figures 9(a) and 9(b) are Lyapunov spectrums of the channel 9 and 10 after being filtered 0–45 Hz. The Lyapunov coefficients start decreasing around second 700 and reach minimum around second 725. There is the interval in that pre-seizure and onset seizure occurs. The minimum of value *λ*_1_ is used for detecting seizure. Moreover, values of *λ*_1_ in both channels have peaks when time of seizure happens. This showed that estimating the spectrum of several Lyapunov exponents (including positive, zeros, and even negative ones) is necessary for quantifing many physical quantities, especially for complicating EEG signals.

 For the sets of the scalp EEG (see Table 1), 8 cases of general epilepsy were not only detected but also replaced by the combination of ICA and the Lyapunov exponent (includes the largest Lyapunov exponent and the Lyapunov spectrum) method. It means that ICA algorithm successfully separates the seizure signal within the brain. Both the largest Lyapunov exponents and Lyapunov spectrum can quantify the nonlinear changes in brain dynamic. Besides, all 8 data sets showed that the Lyapunov spectrum can detect the seizure while the largest Lyapunov exponent cannot do this for the scalp EEG without analysing ICA. This result should be an advantage for processing EEG signal.

## 4. Conclusions

A proposal for the estimation of Lyapunov spectrum profiles from EEG to diagnose the epilepsy has been presented. The results of the experiments clearly show that the proposal carried out advantages than the combination of ICA and the largest Lyapunov exponent method. The ICA algorithm successfully separated the seizure signal from the rest of the sources, noise, and artifacts within the brain and the largest Lyapunov exponent evaluated the chaotic behavior of the EEG signals. Lyapunov spectrum is considered as a robust and general method to process EEG signal to detect epilepsy. The results obtained for the estimated source are similar to diagnosis from medical doctors in case of typical general epilepsy.

## Figures and Tables

**Table 1:** Characteristics of the recordings (obtained in the Department of Clinical neurophysiology at Hospital 115 in Vietnam).

Type of epilepsy	No. of patients Males/females	Age ranges	Recording length ranges (mins)	No. of electrodes
General epilepsy	7/1	30–45	20–30	22
